# The genome sequence of the chicken of the woods fungus, *Laetiporus sulphureus *(Bull.) Murrill, 1920

**DOI:** 10.12688/wellcomeopenres.17750.1

**Published:** 2022-03-09

**Authors:** Richard Wright, Kieran Woof, Brian Douglas, Ester Gaya, Royal Botanic Gardens Kew Genome Acquisition Lab, Darwin Tree of Life Barcoding collective, Wellcome Sanger Institute Tree of Life programme, Wellcome Sanger Institute Scientific Operations: DNA Pipelines collective, Tree of Life Core Informatics collective, Darwin Tree of Life Consortium

**Affiliations:** 1 Royal Botanic Gardens, Kew, Richmond, Surrey, UK; 2 School of Biosciences, Cardiff University, Cardiff, UK

**Keywords:** Laetiporus sulphureus, chicken of the woods, genome sequence, chromosomal, Fungi

## Abstract

We present a genome assembly from an individual *Laetiporus sulphureus* (the chicken of the woods fungus; Basidiomycota; Agaricomycetes; Polyporales; Laetiporaceae). The genome sequence is 37.4 megabases in span. The complete assembly is scaffolded into 14 chromosomal pseudomolecules.

## Species taxonomy

Eukaryota; Opisthokonta; Fungi; Dikarya; Basidiomycota; Agaricomycotina; Agaricomycetes; incertae sedis; Polyporales; Laetiporaceae; *Laetiporus*; *Laetiporus sulphureus* ((Bull.) Murrill, 1920) (NCBI:txid5630)

## Background

*Laetiporus sulphureus* (chicken of the woods) is a poroid fungus that produces large, fleshy, orange to citric yellow, annual bracket-like sporocarps, with an undulating margin, which can grow up to 40 cm in size. A smooth, vivid yellow pore layer with 3–4 pores per millimetre can be observed in the underside. Sporocarps can occur individually or in large imbricate clusters. They have a dimitic hyphal structure and the generative hyphae lack clamps. Spores are ellipsoid to ovoid, smooth, hyaline, measuring 5–8 × 4–5 μm ( Ryvarden & Melo, 2017). In culture, the hyphae are hyaline, clamped and sparingly branched, with some aerial growth. They produce large, thick-walled, terminal chlamydospores and smaller thin-walled conidia. Aging cultures appear dull yellow or orange, with a granular texture due to asexual spore production. 

This species can form dense, vertical, rubbery sheets of mycelium along radial lines in well colonised heartwood, which are a distinctive identification feature when sporocarps are not present.

*Laetiporus sulphureus* has a distribution that includes Europe as well as North and South America ( Song & Cui, 2017). Throughout Europe it is known to cause a brown-rot in the heartwood of a range of angiosperm trees, particularly *Quercus*, where it is a key engineer of hollowing, but it can also be found in species of *Castanea, Fraxinus, Prunus*, and *Salix*. It is also considered to occur on the gymnosperm *Taxus baccata*. In the UK, *L . sulphureus* is widespread and common wherever host trees are present.

*Laetiporus sulphureus* represents a group of cryptically diverse taxa that in recent years has undergone significant and ongoing revision ( Song *et al.*, 2018). The genome produced for this collection has the potential to help us resolve the cryptic diversity of this genus in the UK and to greatly expand our understanding of how these essential wood recyclers operate and interact in their environment. This information may be critical to our ability to protect the associated organisms that rely on dead wood and tree hollow habitats.

## Genome sequence report

**Figure 1.**
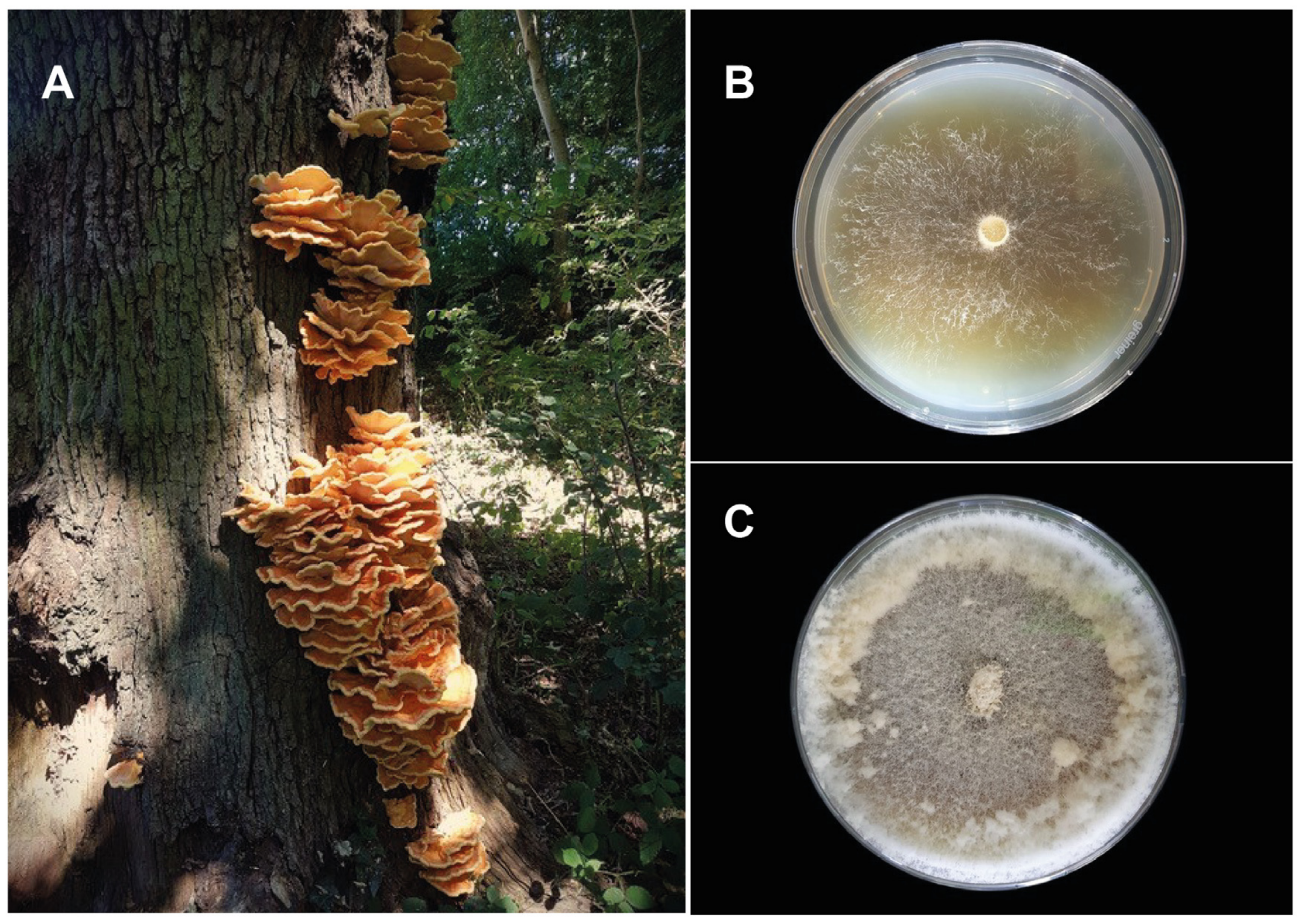
Images of the *Laetiporus sulphureus* specimen used for genome sequencing. ( **A**) *Laetiporus sulphureus* spore producing bodies from which the culture for this genome was isolated, growing on the trunk of *Quercus robur* at Oldbury Court, Bristol. ( **B**) An isolate of *L. sulphureus* at 10 days growth on 0.5% malt tannic acid agar. ( **C**) An isolate of L. sulphureus at 30 days growth on 2 % malt extract agar.

The genome was sequenced from a single *L. sulphureus* specimen ( Figure 1) collected from Oldbury Court, Bristol, UK (latitude 51.4897, longitude -2.5253). A total of 106-fold coverage in Pacific Biosciences single-molecule HiFi long reads and 1853-fold coverage in 10X Genomics read clouds were generated. Primary assembly contigs were scaffolded with chromosome conformation Hi-C data. Manual assembly curation corrected 7 missing/misjoins and removed 3 haplotypic duplications, reducing the assembly size by 2.87%, the scaffold number by 36.36% and the scaffold N50 by 0.88%.

**Figure 2.**
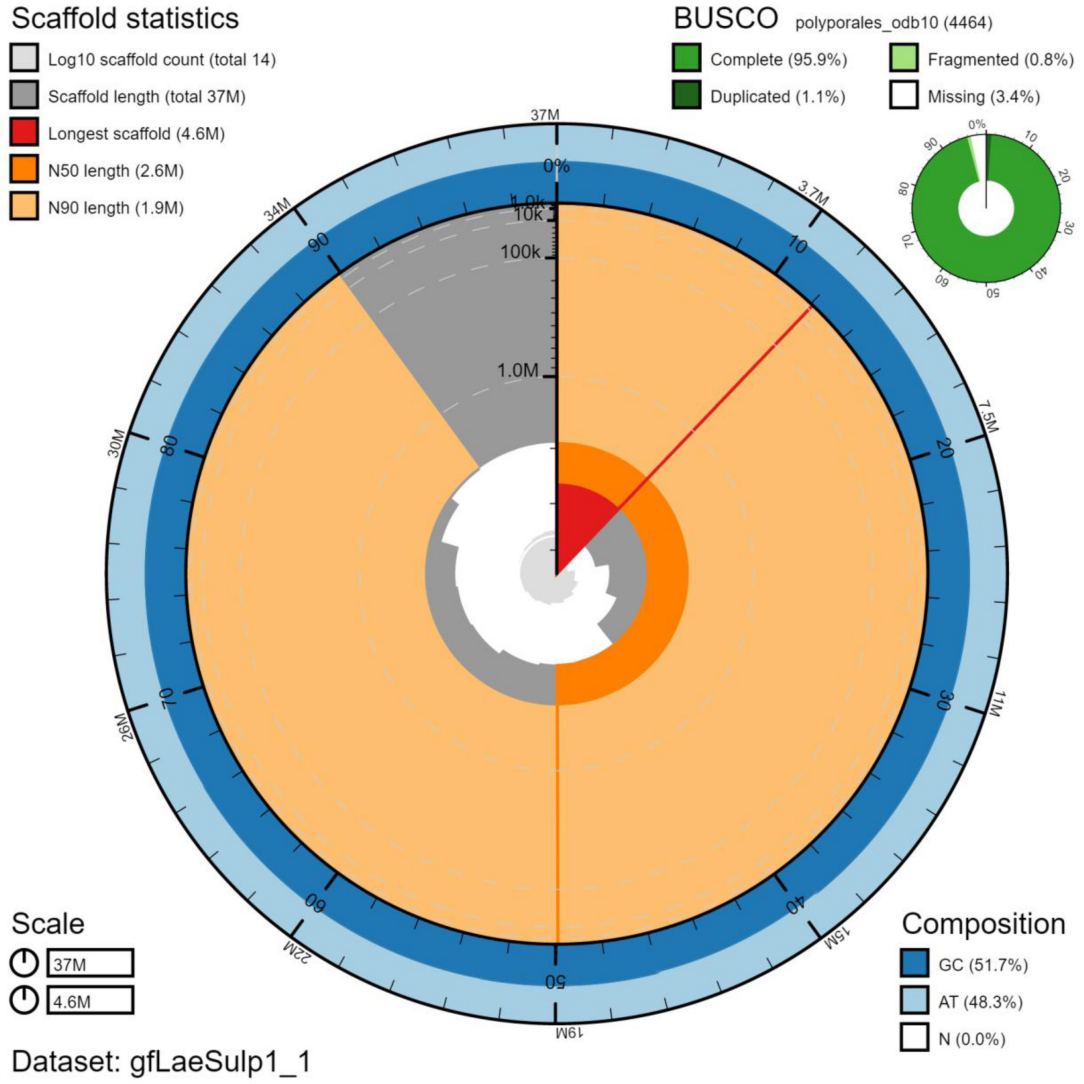
Genome assembly of *Laetiporus sulphureus*, gfLaeSulp1.1: metrics. The main plot is divided into 1,000 size-ordered bins around the circumference with each bin representing 0.1% of the 37,410,216 bp assembly. The distribution of chromosome lengths is shown in dark grey with the plot radius scaled to the longest chromosome present in the assembly (4,563,458 bp, shown in red). Orange and pale-orange arcs show the N50 and N90 chromosome lengths (2,582,225 and 1,895,436 bp), respectively. The pale grey spiral shows the cumulative chromosome count on a log scale with white scale lines showing successive orders of magnitude. The blue and pale-blue area around the outside of the plot shows the distribution of GC, AT and N percentages in the same bins as the inner plot. A summary of complete, fragmented, duplicated and missing BUSCO genes in the polyporales_odb10 set is shown in the top right. An interactive version of this figure is available at https://blobtoolkit.genomehubs.org/view/gfLaeSulp1_1/dataset/gfLaeSulp1_1/snail.

The final assembly has a total length of 37.4 Mb in 14 sequence scaffolds with a scaffold N50 of 2.6 Mb ( Table 1). Of the assembly sequence, 100% was assigned to 14 chromosomal-level scaffolds (numbered by sequence length) ( Figure 2– Figure 5; Table 2). The assembly has a BUSCO ( Manni *et al.*, 2021) completeness of 95.9% (single, 94.8%, duplicated 1.1%) using the polyporales_odb10 reference set (n=4464). While not fully phased, the assembly deposited is of one haplotype. Contigs corresponding to the second haplotype have also been deposited.

**Table 1.** Genome data for *Laetiporus sulphureus*, gfLaeSulp1.1.

*Project accession data*	
Assembly identifier	gfLaeSulp1.1
Species	*Laetiporus sulphureus*
Specimen	gfLaeSulp1
NCBI taxonomy ID	5630
BioProject	PRJEB47319
BioSample ID	SAMEA8562046
Isolate information	Mycelium
*Raw data accessions*	
PacificBiosciences SEQUEL II	ERR6808041
10X Genomics Illumina	ERR6688736-ERR6688739
Hi-C Illumina	ERR6688740
*Genome assembly*	
Assembly accession	GCA_927399515.1
*Accession of alternate haplotype*	GCA_927399535.1
Span (Mb)	37.4
Number of contigs	17
Contig N50 length (Mb)	2.6
Number of scaffolds	14
Scaffold N50 length (Mb)	2.6
Longest scaffold (Mb)	4.6
BUSCO * genome score	C:95.9%[S:94.8%,D:1.1%],F:0.8%,M:3.4%,n:4464

*BUSCO scores based on the polyporales_odb10 BUSCO set using v5.1.2. C= complete [S= single copy, D=duplicated], F=fragmented, M=missing, n=number of orthologues in comparison. A full set of BUSCO scores is available at https://blobtoolkit.genomehubs.org/view/gfLaeSulp1_1/dataset/gfLaeSulp1_1/busco.

**Figure 3.**
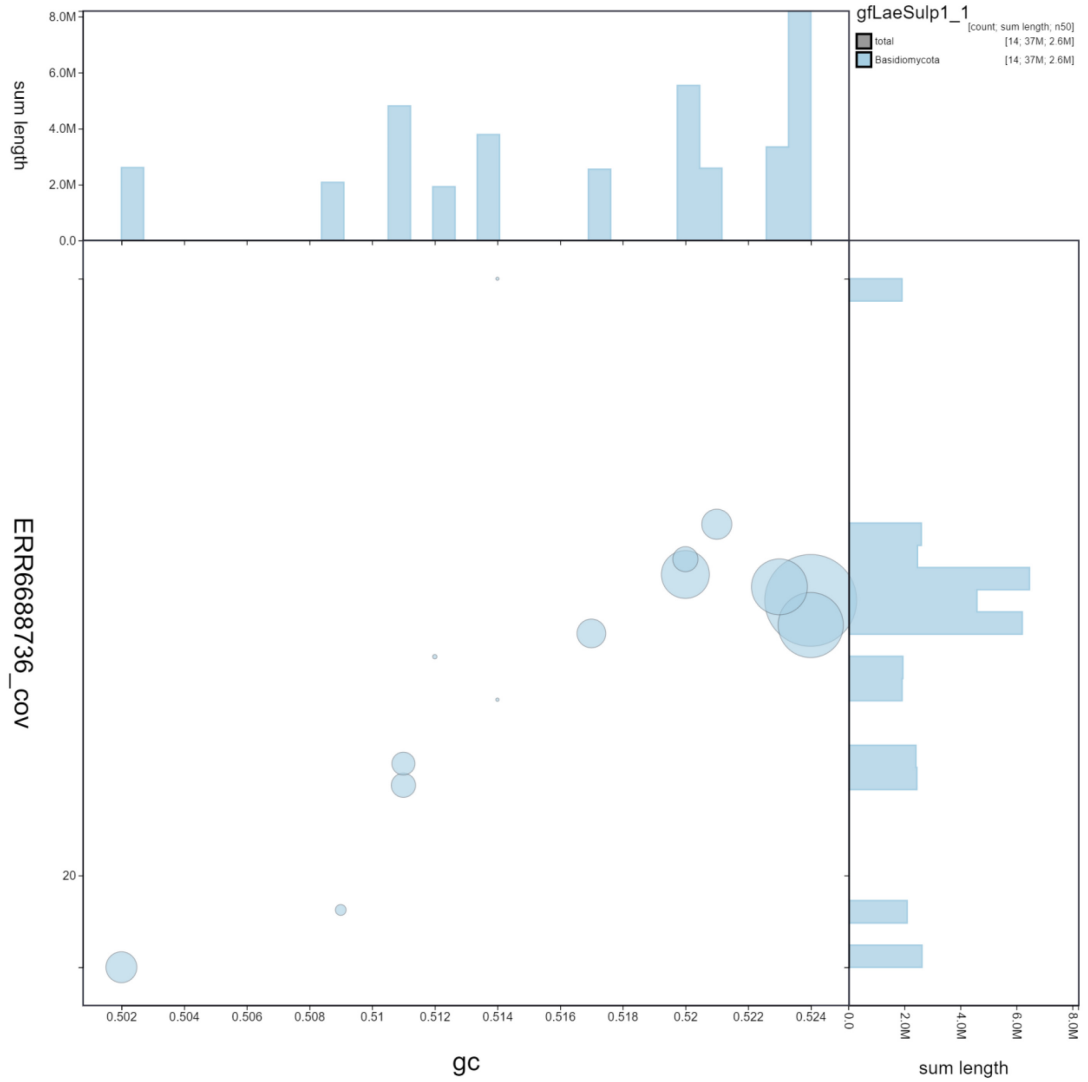
Genome assembly of *Laetiporus sulphureus*, gfLaeSulp1.1: GC coverage. BlobToolKit GC-coverage plot. Scaffolds are coloured by phylum. Circles are sized in proportion to scaffold length. Histograms show the distribution of scaffold length sum along each axis. An interactive version of this figure is available at https://blobtoolkit.genomehubs.org/view/gfLaeSulp1_1/dataset/gfLaeSulp1_1/blob.

**Figure 4.**
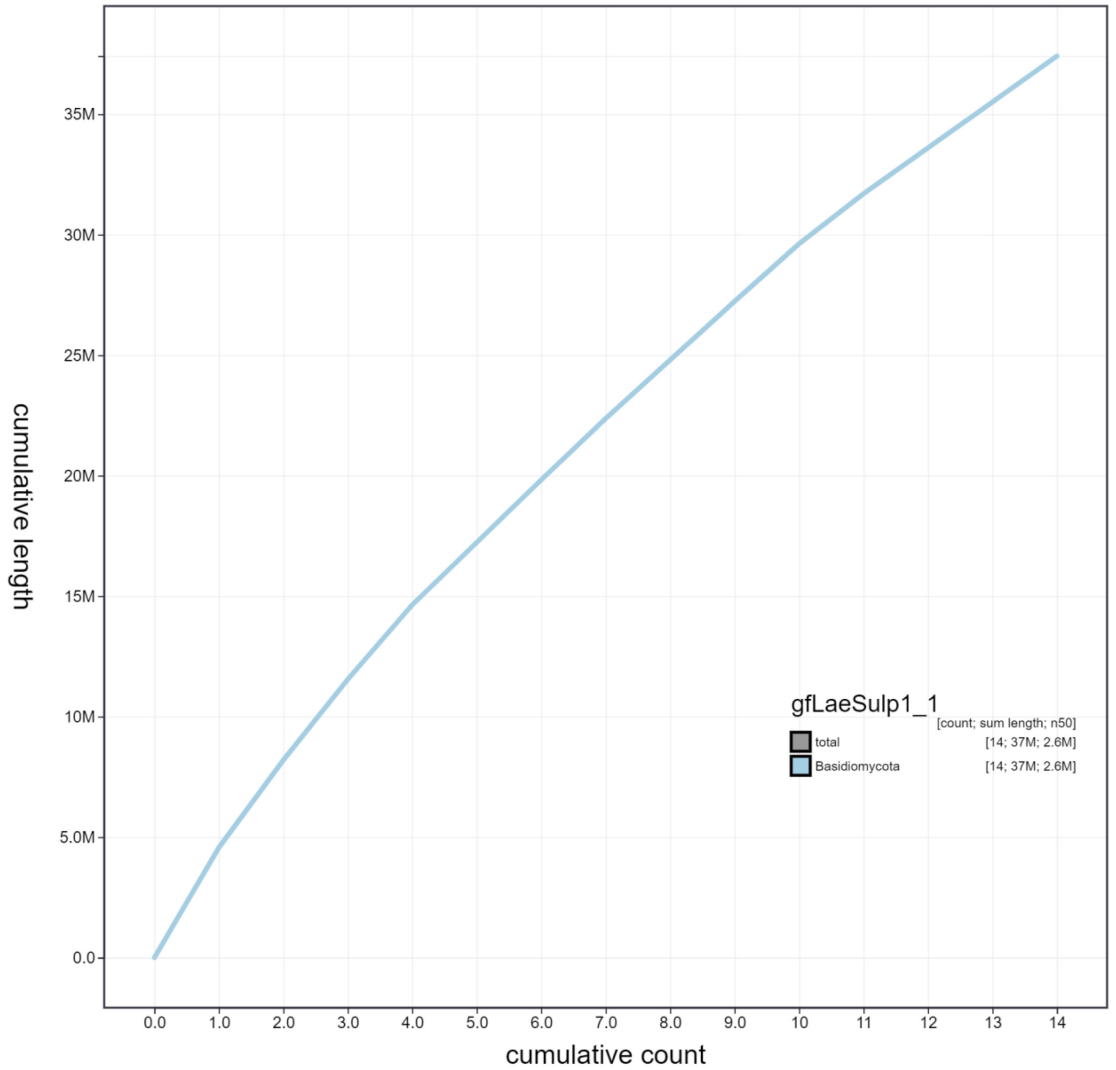
Genome assembly of *Laetiporus sulphureus*, gfLaeSulp1.1: cumulative sequence. BlobToolKit cumulative sequence plot. The grey line shows cumulative length for all scaffolds. Coloured lines show cumulative lengths of scaffolds assigned to each phylum using the buscogenes taxrule. An interactive version of this figure is available at https://blobtoolkit.genomehubs.org/view/gfLaeSulp1_1/dataset/gfLaeSulp1_1/cumulative.

**Figure 5.**
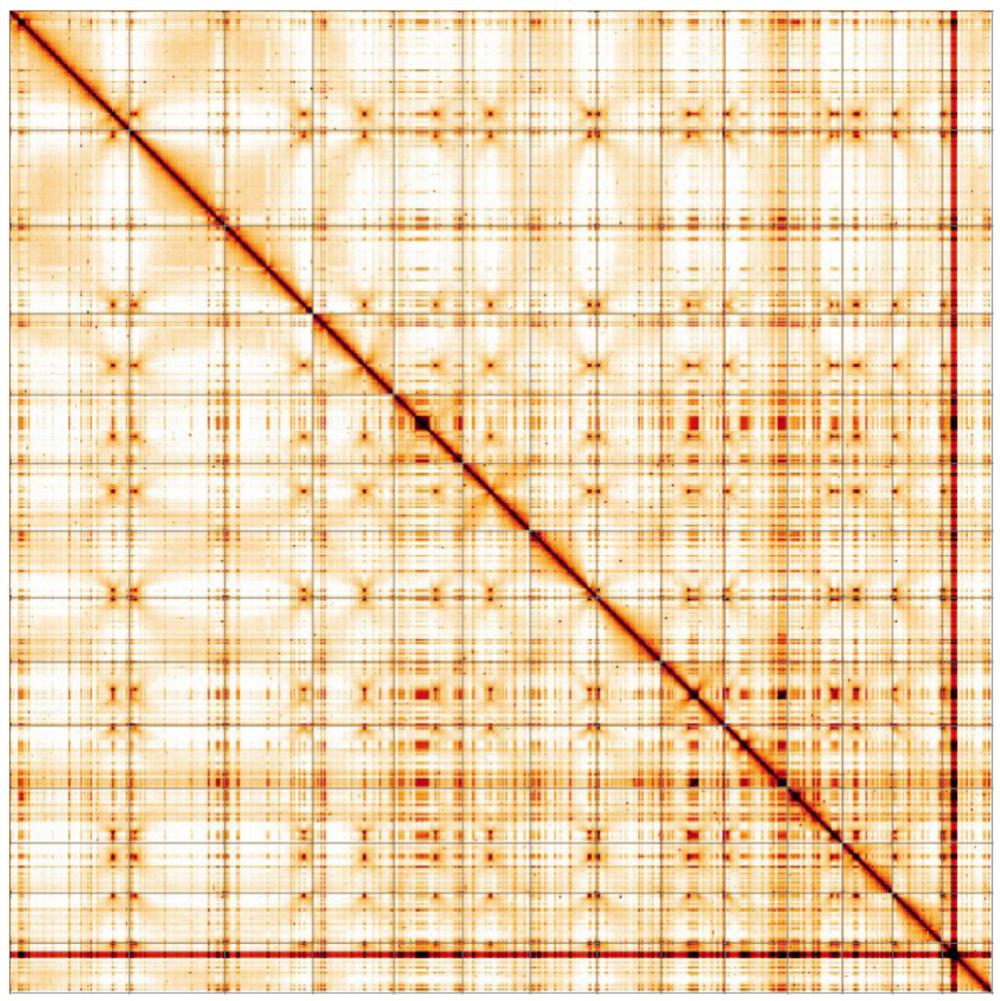
Genome assembly of *Laetiporus sulphureus*, gfLaeSulp1.1: Hi-C contact map. Hi-C contact map of the gfLaeSulp1.2 assembly, visualised in HiGlass. Chromosomes are given in size order from left to right and top to bottom.

**Table 2.** Chromosomal pseudomolecules in the genome assembly of *Laetiporus sulphureus*, gfLaeSulp1.1.

INSDC accession	Chromosome	Size (Mb)	GC%
OV656674.1	1	4.56	52.4
OV656675.1	2	3.64	52.4
OV656676.1	3	3.34	52.3
OV656677.1	4	3.10	52.0
OV656678.1	5	2.61	50.2
OV656679.1	6	2.58	52.1
OV656680.1	7	2.54	51.7
OV656681.1	8	2.44	52.0
OV656682.1	9	2.42	51.1
OV656683.1	10	2.39	51.1
OV656684.1	11	2.08	50.9
OV656685.1	12	1.92	51.2
OV656686.1	13	1.90	51.4
OV656687.1	14	1.89	51.4

## Methods

### Sample acquisition and nucleic acid extraction

A *L. sulphureus specimen* (glLaeSulp1) was collected from Oldbury Court, Bristol, UK (latitude 51.4897, longitude -2.5253) by Richard Wright, Royal Botanic Gardens Kew, from a *Quercus* sp. trunk. The specimens were identified by the same individual and snap-frozen on dry ice.

DNA was extracted at the Tree of Life laboratory, Wellcome Sanger Institute. The gfLaeSulp1 sample was weighed and dissected on dry ice with tissue set aside for Hi-C sequencing. Mycelium tissue was cryogenically disrupted to a fine powder using a Covaris cryoPREP Automated Dry Pulveriser, receiving multiple impacts. Fragment size analysis of 0.01–0.5 ng of DNA was then performed using an Agilent FemtoPulse. High molecular weight (HMW) DNA was extracted using the Qiagen Plant MagAttract HMW DNA extraction kit. Low molecular weight DNA was removed from a 200-ng aliquot of extracted DNA using 0.8X AMpure XP purification kit prior to 10X Chromium sequencing; a minimum of 50 ng DNA was submitted for 10X sequencing. HMW DNA was sheared into an average fragment size between 12–20 kb in a Megaruptor 3 system with speed setting 30. Sheared DNA was purified by solid-phase reversible immobilisation using AMPure PB beads with a 1.8X ratio of beads to sample to remove the shorter fragments and concentrate the DNA sample. The concentration of the sheared and purified DNA was assessed using a Nanodrop spectrophotometer and Qubit Fluorometer and Qubit dsDNA High Sensitivity Assay kit. Fragment size distribution was evaluated by running the sample on the FemtoPulse system.

### Sequencing

Pacific Biosciences HiFi circular consensus and 10X Genomics Chromium read cloud sequencing libraries were constructed according to the manufacturers’ instructions. Sequencing was performed by the Scientific Operations core at the Wellcome Sanger Institute on Pacific Biosciences SEQUEL II (HiFi) and Illumina NovaSeq 6000 (10X) instruments. Hi-C data were generated in the Tree of Life laboratory from mycelium tissue of gfLaeSulp1 using the Arima v2 kit and sequenced on a NovaSeq 6000 instrument.

### Genome assembly

Assembly was carried out with Hifiasm ( Cheng *et al.*, 2021). Haplotypic duplication was identified and removed with purge_dups ( Guan *et al.*, 2020). One round of polishing was performed by aligning 10X Genomics read data to the assembly with longranger align, calling variants with freebayes ( Garrison & Marth, 2012). The assembly was then scaffolded with Hi-C data ( Rao *et al.*, 2014) using SALSA2 ( Ghurye *et al.*, 2019). The assembly was checked for contamination and corrected using the gEVAL system ( Chow *et al.*, 2016) as described previously ( Howe *et al.*, 2021). Manual curation was performed using gEVAL, HiGlass ( Kerpedjiev *et al.*, 2018) and Pretext. The mitochondrial genome was assembled using MitoHiFi ( Uliano-Silva *et al.*, 2021), which performed annotation using MitoFinder ( Allio *et al.*, 2020). The genome was analysed and BUSCO scores generated within the BlobToolKit environment ( Challis *et al.*, 2020). Table 3 contains a list of all software tool versions used, where appropriate.

**Table 3.** Software tools used.

Software tool	Version	Source
Hifiasm	0.15.3	Cheng *et al.,* 2021
purge_dups	1.2.3	Guan *et al.,* 2020
SALSA2	2.2	Ghurye *et al.,* 2019
longranger align	2.2.2	https://support.10xgenomics.com/genome-exome/software/pipelines/latest/advanced/other-pipelines
freebayes	1.3.1-17-gaa2ace8	Garrison & Marth, 2012
MitoHiFi	2.0	Uliano-Silva *et al.,* 2021
gEVAL	N/A	Chow *et al.,* 2016
PretextView	0.2.x	https://github.com/wtsi-hpag/PretextView
HiGlass	1.11.6	Kerpedjiev *et al.,* 2018
BlobToolKit	2.6.4	Challis *et al.*, 2020

## Data availability

European Nucleotide Archive: Laetiporus sulphureus (chicken of the woods). Accession number PRJEB47319; https://identifiers.org/ena.embl/PRJEB47319.

The genome sequence is released openly for reuse. The *L. sulphureus* genome sequencing initiative is part of the Darwin Tree of Life (DToL) project. All raw sequence data and the assembly have been deposited in INSDC databases. The genome will be annotated and presented through the Ensembl pipeline at the European Bioinformatics Institute. Raw data and assembly accession identifiers are reported in Table 1.

## Author information

Members of the Royal Botanic Gardens Kew Genome Acquisition Lab are listed here: https://doi.org./10.5281/zenodo.6283448.

Members of the Darwin Tree of Life Barcoding collective are listed here: https://doi.org/10.5281/zenodo.5744972.

Members of the Wellcome Sanger Institute Tree of Life programme are listed here: https://doi.org/10.5281/zenodo.6125027.

Members of Wellcome Sanger Institute Scientific Operations: DNA Pipelines collective are listed here: https://doi.org/10.5281/zenodo.5746904.

Members of the Tree of Life Core Informatics collective are listed here: https://doi.org/10.5281/zenodo.6125046.

Members of the Darwin Tree of Life Consortium are listed here: https://doi.org/10.5281/zenodo.5638618.
