# Supplementary material for: The genome sequence of the chicken of the woods fungus, Laetiporus sulphureus (Bull.) Murrill, 1920
Source: Wellcome Open Res. 2022 Mar 9;7:83. [Version 1] doi: 10.12688/wellcomeopenres.17750.1 (PMC10652036; doi:10.12688/wellcomeopenres.17750.1)
Supplement: Peer review history

# Reviewer report: Reviewer response for version 1

**DOI:** 10.21956/wellcomeopenres.19645.r69210

**Published:** 2023-11-15

**Authors:** Dilek Guldemir

**Affiliations:** 1 National Parasitology Reference Laboratory, Saglik Mah, Ankara, Turkey

In the relevant Data Note type study, "A genome assembly from an individual *Laetiporus sulphureus* (the chicken of the woods fungus; Basidiomycota; Agaricomycetes; Polyporales; Laetiporaceae). The genome sequence is 37.4 megabases in span. The complete assembly is scaffolded into 14 chromosomal pseudomolecules" is offered.

 The methods used in the study are sufficient and the data obtained are useful. The study is suitable for publication as "Data Note". However, I recommend that the study be expanded and the number of *Laetiporus sulphureus* samples be increased to ensure regional representation, and that they be presented as the original article by comparing them with global WGS data and the reference genome as a representation of the species.

Are sufficient details of methods and materials provided to allow replication by others?

Yes

Is the rationale for creating the dataset(s) clearly described?

Yes

Are the datasets clearly presented in a useable and accessible format?

Yes

Are the protocols appropriate and is the work technically sound?

Yes

Reviewer Expertise:

Medical microbiology, molecular microbiology, pathogens, genetics, bioinformatics, parasitology

I confirm that I have read this submission and believe that I have an appropriate level of expertise to confirm that it is of an acceptable scientific standard.

# Reviewer report: Reviewer response for version 1

**DOI:** 10.21956/wellcomeopenres.19645.r68115

**Published:** 2023-10-09

**Authors:** Miguel Naranjo-Ortiz

**Affiliations:** 1 University of Oslo, Oslo, Norway

The paper describes the protocols used to generate the reference genome of the fungus *Laetiporus sulphureus*. I consider that the methodology and results are sufficiently detailed.

 There is a few very minor and optional suggested additions:

1. I would suggest mentioning in the background that this species is a choice edible mushroom.
2. Since there have been some taxonomic revision, it might be pertinent to disclose recent changes and perhaps even a rationale supporting that the sampled specimen corresponds to the species. For example, because it has a similar geographical range than the type specimen.
3. Ideally the specimen used for data production or a culture derived from it would be available in a biological repository, be it a fungarium, a culture collection or a DNA bank. If that's the case, it should be mentioned.

Are sufficient details of methods and materials provided to allow replication by others?

Yes

Is the rationale for creating the dataset(s) clearly described?

Yes

Are the datasets clearly presented in a useable and accessible format?

Yes

Are the protocols appropriate and is the work technically sound?

Yes

Reviewer Expertise:

Comparative genomics of fungi

I confirm that I have read this submission and believe that I have an appropriate level of expertise to confirm that it is of an acceptable scientific standard.
